# Assessment of Neurodegenerative Changes in Turkeys Fed Diets with Different Proportions of Arginine and Methionine Relative to Lysine

**DOI:** 10.3390/ani12121535

**Published:** 2022-06-14

**Authors:** Magdalena Krauze, Katarzyna Ognik, Dariusz Mikulski, Jan Jankowski

**Affiliations:** 1 Department of Biochemistry and Toxicology, University of Life Sciences, 20-950 Lublin, Poland; katarzyna.ognik@up.lublin.pl; 2 Department of Poultry Science, University of Warmia and Mazury in Olsztyn, 10-719 Olsztyn, Poland; dariusz.mikulski@uwm.edu.pl (D.M.); janj@uwm.edu.pl (J.J.)

**Keywords:** turkey, amino acid, neurodegeneration, methionine, lysine, arginine

## Abstract

**Simple Summary:**

It is important to take care of a properly balanced amino acid composition in the diet in order to inhibit or delay the occurrence of processes and changes related to the destruction of nervous tissue. Therefore, an attempt was made in this manuscript to evaluate the effect of different ratios of the key amino acids arginine and methionine, relative to lysine, in relation to two turkey feeding standards. The amino acid guidelines formulated by British United Turkeys (BUT) suggest higher levels of lysine (Lys) in turkey diets than those recommended by the National Research Council (NRC). In order to assess the impact of such supplementation, we analyzed the level of indicators informing the presence or degree of advancement of neurodegenerative processes in the nervous tissue (the level of acetylcholinesterase and amyloid-β; the concentration of AChE complexes with amyloid-β and Tau protein, called glycosylated acetylcholinesterase (GAChE), indicative of the destruction of neurons). The level of low-density lipoprotein receptor-related protein 1, or LRP-1, which facilitates the breakdown of toxic amyloid-β, was also assessed. In addition, the effect of different doses of these amino acids on neurodegenerative changes in DNA, especially the degree of methylation of histone proteins resulting from covalent modifications was compared between lysine and arginine residues.

**Abstract:**

We postulated that the use of optimal levels and proportions of Arg and Met relative to a low or high concentration of Lys in diets for meat turkeys would reduce the occurrence of metabolic disturbances in the nervous tissue that can lead to neurodegenerative changes. The aim of the study was to determine the effect of various proportions of Lys, Arg, and Met in diets for turkeys, with a low content of Lys in accordance with NRC (Experiment 1) recommendations, and in diets with high Lys levels that are close to the recommendations of breeding companies (Experiment 2) on selected indicators of potential neurodegenerative effects in the brain and liver of turkeys. The Experiment 1 and Experiment 2 was conducted using 864 day-old turkey chicks randomly assigned to six groups, in eight replicates (6 groups × 18 birds × 8 replicates). A full description of the methodology can be found in previously published papers using the same experimental design. Indicators informing about the presence or advancement of neurodegenerative processes in the nervous tissue were determined in the brain and liver (level of: AChE, amyloid-β, GAChE, Tau protein, LRP1, and the degree of DNA methylation). It was established that in the case of both a low (National Research Council, NRC) and a high (British United Turkeys, BUT) level of Lys in the diet of turkeys, the Arg level can be reduced to 90% of the Lys level and Met to 30% of the Lys level, because this does not cause neurodegenerative changes in turkeys. Unfavorable neurodegenerative changes may appear if the Arg level is increased from 100 to 110% of the Lys level recommended by the NRC. However, due to the lack of such a relationship when Arg is increased from 100 to 110% of the Lys level recommended by BUT, at this stage of research no definitive conclusions can be drawn regarding the risk of neurodegenerative changes caused by increasing Arg in the diet of turkeys.

## 1. Introduction

A diet with an appropriate amino acid profile plays a key role in exploiting the genetic potential of fast-growing turkeys [1]. The main amino acids limiting the biological value of dietary protein in poultry are methionine (Met) and arginine (Arg) [2,3]. Lysine (Lys), on the other hand, may determine the digestibility and absorption of Arg in the intestines [4]. Requirements for the intake of amino acids in the diet of turkeys formulated by NRC, as seen in [5,6], differ substantially in the proportions of individual amino acids. According to the NRC [5], the Arg level in the diet of turkeys should be 90–100% of the Lys content, whereas BUT [6] recommends a higher Arg level (102–105% Lys). The Met level established by the NRC [5] is 30–38% of the content of Lys, while BUT [6] recommends a level of 36–41%. Supplementation of the diet with selected amino acids can have highly varied effects on the functioning of the brain, liver, and nervous tissue, and the biosynthesis of amino acids in the brain and liver is strictly regulated by the concentration of amino acids in the plasma [7]. According to Morales et al. [8], optimal levels of amino acids can play a significant role in maintaining the integrity and functioning of brain cells and hepatocytes, take part in the synthesis of neurotransmitters, and—owing to their ability to regenerate and reorganize neurons—play an active role in the treatment of neurological diseases. Excessive amounts of these compounds, on the other hand, can have toxic effects on nervous tissue, disturb its metabolism, and lead to neurodegenerative changes [7,9,10].

According to Toue et al. [11] and Koladiya et al. [12], one of the amino acids essential to the normal functioning of nervous tissue is methionine. Methionine and cysteine, which is derived from it, are key amino acids with an important role in the cellular metabolism of neurons. Some other authors report that excessive methionine intake in the diet may induce neurodegenerative changes in the brain and accelerate the ageing process, which may be caused by methionine sulfoxide, an intermediate product of the methionine cycle [7,13,14,15,16]. On the other hand, according to Toue et al. [11], a deficiency of methionine can cause dementia. In addition, excessive amounts of cysteine, derived from methionine, can also have neurotoxic effects. The most neurotoxic form of cysteine, S-nitroso cysteine, is responsible for inhibition of mitochondrial respiration, caused by metabolic disturbances and inhibition of ATP synthesis in the neurons. This compound is produced in a reaction between methionine and reactive oxygen species, mainly nitric oxide (NO) [17]. Another amino acid with an important role in determining the biological value of fodder protein is arginine (Arg). There is no urea cycle in birds, and as a result, Arg is not synthesized endogenously, therefore, it must be added to their diet [18].

According to Rajagopal et al. [19], Arg stimulates nerve signal transmission between neurons, activates the growth and differentiation of new nerve cells, and delays neuron apoptosis. According to Balnave and Brake [4], both the deficiency and surplus of Arg can adversely affect the concentrations of other amino acids in the blood plasma and muscles, which impedes not only nerve cell metabolism but also the growth and development of birds. This is because Arg is a precursor of NO, ornithine, glutamate, creatine, proline, glutamine, and dimethylarginine, and therefore plays an important role in the metabolism of birds [20].

Lysine is another amino acid that is essential for the growing turkeys, and a Lys deficiency or defects associated with catabolism of this amino acid can result in neurodegenerative and other physiological changes [21]. Nutritional studies conducted by Jankowski et al. [2,3] and Ognik et al. [22] have shown that a diet with low Lys content, recommended by the NRC [5], with an Arg level of 100% relative to Lys and a Met level of 45% of the Lys level, can stimulate immune and antioxidant defenses, as well as eliminate oxidative changes and neutralize biologically important compounds. Another study by the same authors showed that in growing turkeys, which were fed a diet with a high Lys content (according to the recommendations of BUT [6], Arg and Met can reach levels of 90 and 45% of the Lys content with no negative effect on metabolic parameters or the growth of the birds [23]). In addition, the inclusion of Met at a level of 45% of Lys levels increases the antioxidant potential of turkeys and reduces the risk of oxidative changes in important biomolecules, especially lipids and proteins, as well as DNA.

Diets for turkeys with a high Arg content (110% Lys) were found to be unfavorable, due to the risk of lipid oxidation, protein nitration, and adverse changes in the concentrations of hormones regulating metabolism, especially of sugars [22]. Due to the potential neurotoxic effects of a diet with excessive Met, which must be correctly correlated with Arg, relative to Lys [5,6], in order to choose the optimal levels of these amino acids, it seems important to determine indicators of the appearance of neurodegenerative changes in the brain resulting from the use of these amino acids. For this purpose, it may be useful to analyze the level of acetylcholinesterase (AChE), a key enzyme for conduction of nerve impulses and protection of neurons, as well as the levels of neurotoxic amyloid-β and the concentration of AChE complexes with amyloid-β and Tau protein, called glycosylated acetylcholinesterase (GAChE), which are responsible for neuronal apoptosis. Both a deficiency of acetylcholinesterase and the formation of complexes of this enzyme with amyloid-β (glycosylated acetylcholinesterase, GAChE) are conducive to mitochondrial dysfunction and the generation of large amounts of reactive oxygen species, which initiate neuronal apoptosis [24,25,26,27]. Moreover, it may be useful to determine the level of low-density lipoprotein receptor-related protein 1 (LRP1), which facilitates the degradation of toxic amyloid-β [28]. Evaluation of the degree of methylation of histone proteins, resulting from the covalent modifications of lysine and arginine residues, may also be helpful in recognizing the intensification of neurodegenerative changes [29].

A further suggestion to explore the question of the effect of different levels and proportions of lysine, arginine, and methionine in diets for meat turkeys was the findings of other researchers, who evaluated the rearing efficiency, as well as biochemical and immune parameters of the blood, intestinal morphology, dressing percentage, and meat quality [30,31,32]. According to Ghamari Monavvar et al. [33], additional supplementation with arginine improves growth performance and the quality traits of poultry (especially meat tenderness and meat fat content). What is more, Arg supplementation improves the intestinal morphology (especially the ratio of villus height to crypt depth), enzyme activity, and the composition of the intestinal microbiota. It also stimulates the immune system, a significant portion of which is located in the intestines, and increases levels of insulin and thyroid hormones, thus activating metabolism. A new aspect of the study is focused on the problem of choosing optimum levels and proportions of Arg and Met relative to a low or high concentration of Lys in diets for meat turkeys, in the context of minimizing the occurrence of metabolic disorders in nervous tissue. Efforts to improve rearing efficiency in turkeys by modifying their diet may adversely affect health parameters, disturb the general metabolism, increase the risk of oxidative stress, and importantly, affect nervous tissue as well.

We found no reports in the world literature of research conducted in poultry in the context of neurodegenerative changes. Because Arg, Met, and Lys are essential amino acids for poultry and determine the biological value of fodder protein, it seems useful to observe such changes, especially when we attempt to improve the growth performance of turkeys by changing the proportions of these amino acids. We took an interest in potential neurodegenerative changes after an in-depth analysis of the results obtained by the co-authors of this study in their research on growth performance and the indicators of sugar metabolism, peptide nitration, lipid oxidation, and systemic antioxidant defenses [2,3,22,23].

Due to the fact that the nervous tissue plays a superior role in the functioning of the system, any interference leading to the improvement of its condition is justified, provided that the portion of amino acids used does not constitute a toxic dose, and we tried to avoid this problem by assessing the impact of individual proportions within the acceptable standards for poultry. We considered that, due to the fact that two different nutritional standards for poultry (NRC, BUT) that are commonly used in poultry farming give slightly different values for the nutritional recommendations of Arg, Met, and Lys, and that these amino acids limit the biological value of the dietary protein in poultry and may affect the metabolism of nervous tissue in a different way, it is important to balance them carefully and in our opinion there is a need for a thorough verification of this problem.

We postulated that the use of optimal levels and proportions of Arg and Met relative to a low or high concentration of Lys in diets for meat turkeys would reduce the occurrence of metabolic disturbances in the nervous tissue that can lead to neurodegenerative changes. The aim of the study was to determine the effect of various proportions of Lys, Arg, and Met in diets for turkeys with a low content of Lys, in accordance with NRC [5] recommendations, and in diets with high Lys levels, close to the recommendations of breeding companies [6], on selected indicators of potential neurodegenerative effects in the brain and liver of turkeys.

## 2. Materials and Methods

### 2.1. Experiment 1

The experiment was conducted using 864 day-old turkey chicks randomly assigned to 6 groups, in 8 replicates (6 groups × 18 birds × 8 replicates). A full description of the methodology can be found in previously published papers using the same experimental design [2,3,23,30]. During each of 4 feeding periods (4 weeks each), the birds received ad libitum isocaloric diets containing 1.60, 1.50, 1.30, and 1.00% Lys, in accordance with recommendations for turkeys specified in Nutrient Requirements of Poultry [5]. The factors differentiating the experimental groups were the level of Arg, which was 90, 100, and 110% of the level of Lys in the diet, and for Met it was 30 or 45% of the level of Lys in the diet. For each of the 4 feeding periods, basal diets were prepared without the addition of Lys, Met, or Arg (Table 1). The content of amino acids in the basal diets was determined (Table 2), and then they were mixed with the appropriate amounts of Lys, Met, and Arg. The total content of amino acids in all experimental diets was determined analytically (Table 2). Starter diets (days 1–28) and grower and finisher diets (days 29–112), with no feed additives, were provided as crumbles and pellets (3 mm pellets at 65 °C for 45 s), respectively.

### 2.2. Experiment 2

The experiment was conducted using 864 day-old turkey chicks, which were randomly assigned to 6 groups, in 8 replicates (6 groups × 18 birds × 8 replicates). A full description of the methodology can be found in previously published papers using the same experimental design [3,24]. During each of 4 feeding periods (4 weeks each), the birds received ad libitum isocaloric diets containing 1.83, 1.67, 1.48, and 1.20% Lys, in accordance with recommendations for turkeys by Hybrid Turkeys [6]. The factors differentiating the experimental groups were the level of Arg, which was 90, 100, and 110% of the level of Lys in the diet, and for Met it was 30 or 45% of the level of Lys in the diet. For each of the 4 feeding periods, basal diets were prepared without the addition of Lys, Met, or Arg (Table 3). The content of amino acids in the basal diets was determined (Table 4), and then they were mixed with the appropriate amounts of Lys, Met, and Arg. The total content of amino acids in all experimental diets was determined analytically (Table 4). Starter diets (days 1–28) and grower and finisher diets (days 29–112), with no feed additives, were provided as crumbles and pellets (3 mm pellets at 65 °C for 45 s), respectively.

### 2.3. Laboratory Analyses

At the end of the experiment the birds were weighed after an 8 h feed deprivation, and one bird from each replicate representing the group average BW was selected and euthanized after electrical stunning. Birds were then hung on a processing line and bled out for 3 min by a unilateral neck cut, severing the right carotid artery and jugular vein. The non-edible viscera, including the intestines, proventriculus, gall bladder, spleen, esophagus, and full crop, were manually excised after scalding at 61 °C for 60 s and defeathering in a rotary drum picker for 25 s. After removing the head, the brain and liver were collected for further analysis.

### 2.4. Determination of Indicators of Potential Neurodegenerative Effects and Epigenetic DNA Damage

The activity of acetylcholinesterase (AChE) was determined in homogenates of the brain tissue (Experiments 1 and 2) and liver tissue (Experiment 2) of turkeys using the Chicken Acetylcholinesterase ELISA Kit (Bioassay Technology Laboratory, Inc., Shanghai, China), and the amyloid-β level was determined using the Chicken Total β amyloid Protein (βAP) ELISA Kit (Blue Gene Biotech, Shanghai, China). The levels of glycosylated acetylcholinesterase (GAChE) in the brain (Experiments 1 and 2) and liver (Experiment 2) of the turkeys were determined using the Chicken Glycosylated Acetylcholinesterase ELISA kit (Blue Gene Biotech, Shanghai, China). Low-density lipoprotein receptor-related protein 1 (LRP1) in the homogenates of brain tissue (Experiments 1 and 2) and liver tissue (Experiment 2) were determined using the Chicken Low Density Lipoprotein Receptor Related Protein 1 ELISA kit (Blue Gene Biotech, Shanghai, China). The level of phosphorylated Tau protein was determined using the Chicken Phosphorylated tau 231 (p Tau231) ELISA kit (Blue Gene Biotech, Shanghai, China). The levels of epigenetic changes in the brain (Experiments 1 and 2) and liver (Experiment 2) were determined by analyzing global DNA methylation (methylome), using diagnostic kits from Sigma Aldrich.

### 2.5. Statistical Analysis

This experiment was performed in a completely randomized 3 × 2 factorial design, and the data (presented as the mean ± standard error of the mean) were subjected to a 2-way ANOVA to examine the effect of 3 levels of Arg (90, 100, and 110%) and 2 levels of Met (30 and 45%). The Shapiro–Wilk and Levene tests were applied to test the model assumptions of normality and homogeneity of variance. When a significant interaction effect was noted (F test), treatment means were separated using the post-hoc Tukey’s test. The significance level was set at *p* < 0.05, and statistical calculations were performed using the GLM procedures of the STATISTICA software system ver. 12.0 (Stat Soft Inc., Tulsa, OK, USA, 2014).

## 3. Results

### 3.1. Effect of Different Levels of Arg and Met Relative to Lys, According to the NRC

Differences in Arg content relative to Lys [5] caused no significant changes in the GAChE level in the brain of turkeys (Table 5). The use of a higher proportion of Arg (110% of the Lys level) in the diet caused an increase in the levels of AChE (*p* = 0.019), amyloid-β (*p* = 0.032), and Tau protein (*p* = 0.002) and a decrease in the level of LRP 1 (*p* = 0.035) in the brain of the turkeys. Decreasing Arg content to 90% of the Lys level caused a decrease in AChE (*p* = 0.019) in the brain. Increasing the Met content from 30 to 45% of the Lys level (NRC, 1994) [5] had no effect on the level of AChE, GAChE, amyloid-β, LRP 1, or Tau protein in the brain (Table 5). In the case of Tau protein, an Arg × Met interaction (*p* = 0.05) was noted: when the intermediate Arg level (100% of the Lys level) was applied, increasing the Met content from 30 to 45% of the Lys level caused an increase in the content of Tau protein, which was not noted in the cases of the lowest and highest Arg levels used (90 and 110% of the Lys level) (Table 5).

### 3.2. Effect of Different Levels of Arg and Met Relative to Lys, According to the BUT

Turkeys that were fed diets with the highest Arg content (110%) relative to Lys [6] had the lowest AChE and GAChE levels (*p* < 0.001, both) and the lowest percentage of DNA methylation (*p* < 0.001) in the brain (Table 6). In comparison to the turkeys receiving diets with the intermediate (100% of the Lys level) and highest (110% of the Lys level) content of Arg, the diet with its lowest content (90% of the Lys level) resulted in an increase in LRP 1 (*p* = 0.047) in the brain. Increasing the Met content from 30 to 45% of the Lys level [6] had no effect on the level of AChE, GAChE, amyloid-β, LRP 1, or Tau protein or on the percentage of DNA methylation in the brain of turkeys (Table 6). In the case of amyloid-β there was also an Arg × Met interaction (*p* = 0.042), as the amyloid-β level in the brain decreased when the Met content was increased from 30 to 45% of the Lys level while maintaining the 1:1 Arg to Lys ratio, which was not observed in the case of the reduced Arg content (90% Lys) or increased Arg content (110% Lys). The two-way ANOVA showed an Arg × Met interaction for Tau protein in the brain (*p* = 0.035); in the case of the lowest Arg level (90% of the Lys level), increasing the Met content from 30 to 45% of the Lys level reduced the level of Tau protein, which was not observed in the case of the intermediate and highest Arg content (100 and 110% of the Lys level, respectively) (Table 6). Differences in Arg content relative to Lys [6] caused no statistically significant changes in the LRP1 or Tau protein levels in the liver of turkeys (Table 7). In comparison with the intermediate Arg content (100% of the Lys level) in the diet, reducing Arg to 90% of the Lys level caused an increase in AChE content (*p* = 0.022) in the liver. The two-way ANOVA showed Arg × Met interactions for GAChE and amyloid-β in the liver (*p* = 0.008, *p* = 0.008, *p* = 0.025; respectively). The interaction for GAChE resulted from the fact that, in the case of the highest Arg content (110% of the Lys level), increasing the Met content from 30 to 45% of the Lys level caused an increase in GAChE in the liver, which was not observed in the case of the intermediate and the lowest levels of Arg (100 and 90% of the Lys level, respectively). In the case of amyloid-β, the interaction resulted from the fact that when the lowest Arg level was applied (110% of the Lys level), increasing the Met content from 30 to 45% of the Lys level caused a decrease in the level of amyloid-β in the liver, which was not observed in the case of the intermediate and lowest levels of Arg (100 and 90% of the Lys level, respectively) (Table 7).

## 4. Discussion

Due to the lack of similar studies in the world literature in animal models or data on human patients, it is difficult to compare our results on neurodegenerative changes in turkeys fed diets with different proportions of arginine and methionine relative to lysine. It is also difficult to explain the more specific mechanism of physiological reactions and changes in the biochemical parameters, due to the diametrically different effects in the two experiments, depending on the level of Arg.

In Experiment 1, where the Lys content of turkey diets was based on NRC [5] guidelines, an increase in Arg and Met inclusion rates to Arg 100% and Met 45% of Lys content, respectively, improved BWG [2]. In the 16th week of rearing, the body weight of the turkeys was very similar between the groups, but the lowest value was recorded for the turkeys from the Arg 90%, Met 30% (10.2 kg) treatment, and the highest for the Arg 90% and Met 45% and also Arg 100% and Met 45% (10.7 kg) treatments. In Experiment 2, differences in Arg and Met inclusion rates in diets, relative to Lys content (which was close to BUT [6] recommendations, i.e., high) did not affect the final BW of turkeys. In the 16th week of rearing, the body weight of the turkeys was very similar between the groups, however, the final body weight was slightly higher than in Experiment 1. The lowest value was recorded for the turkeys from the Arg 100%, Met 45% (11.38 kg) treatment, and the highest was recorded for the Arg 110%, Met 45% (11.49 kg) treatment [3].

Among the pathological changes taking place in neurodegenerative diseases, in both humans and laboratory animals, the first symptoms are the excessive deposition of amyloid-β in the brain and neurofibrillary degeneration in the form of hyperphosphorylated Tau protein. According to Całyniuk et al. [24] Arg promotes growth performance in turkeys because it acts as a substrate for creatine biosynthesis. On the other hand, methionine is also directly implicated in creatine synthesis, because it donates a methyl group to glycocyamine (it is a biological precursor for creatine synthesis in birds) which is synthesized from Arg and Gly [34]. What is more, the neuroprotective effect of creatine is related to the intensification of the processes of neurogenesis, supporting the biosynthesis of serotonin and dopamine, and inhibiting the aggregation of amyloid and Tau protein [35].

According to Li et al. [36], amyloid-β fibers can also accumulate in the liver. Amyloid-β additionally circulates in the plasma, the cerebrospinal fluid, and the brain interstitial fluid [37,38], and amyloid deposits can also accumulate near the blood vessels, impairing the functioning of the blood–brain barrier. Complexes of amyloid-β and highly phosphorylated Tau protein with acetylcholinesterase are toxic as well. These complexes are an inactive, glycosylated form of AChE called glycosylated acetylcholinesterase (GAChE). Another pathological symptom of neurodegenerative diseases is a decreasing level of the protein LRP1 or of AChE itself [39,40,41,42,43]. In our study, the highest proportion of Arg (110%) relative to the Lys level recommended by the NRC [5] caused a beneficial increase in the AChE level in combination with a very unfavorable increase in the levels of amyloid-β and Tau protein, as well as an unfavorable decrease in LRP1. The use of the intermediate level of Arg (100% of the Lys level) in combination with increased Met from 30 to 45% also caused an unfavorable increase in the level of Tau protein [44,45].

The decrease in the AChE level following the use of 90% Arg relative to Lys [5] may suggest the initiation of neurodegenerative changes. According to many researchers, a decrease in the level of this enzyme is an early symptom of neurodegenerative disease, which leads to the accumulation of large amounts of acetylcholine in the synaptic spaces and stimulates hyperphosphorylation of Tau protein, which is toxic [46]. A 110% share of Arg relative to Lys [5] cannot be considered to be more beneficial than a 90% share in terms of neurodegenerative changes, despite the increase in the AChE level in the brain tissue. The increase in the AChE level is accompanied by an increase in the level of toxic amyloid-β and Tau protein clusters and a decrease in the level of LRP1 (lipoprotein receptor 1), which is responsible for the removal of amyloid-β from the brain to the blood vessels by active transport [47].

Pathological amyloid-β, produced by fragmentation of its precursor APP (amyloid precursor protein), which is a component of the cell membrane of neurons, is present in tissues in the form of insoluble clusters, and is toxic for nerve cells, called amyloid-β [37,38]. The marked increase in the deposition of amyloid-β in the brain tissue of turkeys receiving a level of Arg that was increased to 110% of the Lys level recommended by NRC [5] is an unfavorable phenomenon. It is likely that excessive Arg stimulates production of amyloid-β, which disturbs calcium balance, damages the mitochondria, and contributes to the release of free radicals that degenerate DNA and cellular proteins. Toxic amyloid-β can cause an increase in the level of Ca^2+^ in the cell, stimulating calmodulin-dependent protein kinase II (CaMKII). Activation of CaMKII in turn stimulates the highly unfavorable phenomenon of hyperphosphorylation of Tau protein. These processes together can lead to oxidative stress, to which nervous tissue is particularly sensitive. This is because neurons use up large amounts of oxygen and have the highest mitochondrial activity of all cells of the body. An increasing amount of research indicates a relationship between oxidative stress of the nervous system and neurodegenerative diseases [48]. In the present study, an excessive amyloid-β deposition in the brain was observed following the use of 110% Arg (relative to the Lys level, recommended by NRC [5], which was not influenced by the Met level. In the liver, the amyloid-β level was lower following the use of 100% Arg and 45% Met (relative to the Lys level, recommended by BUT [6]). The liver plays an important role in removing excess amyloid-β from the blood, which can exacerbate neurodegenerative changes in this organ. In the hepatocytes, amyloid-β is degraded in a reaction with LRP-1, but bile from the liver may prevent this and allow the compound to be excreted unchanged [49,50]. In the nervous tissue of the liver, similarly as in the brain, pathological deposits of proteins (amyloid-β and Tau protein) may accumulate and cause damage to neurons. The presence of significant interactions between Arg and Met supplementation levels in turkey diets with the least Lys (90% of the Lys level, Met 45%) and high Lys (110% of the Lys level, Met 45%) could be attributed to the fact that Tau protein and amyloid-β levels were affected by both Arg and Met content, but in some cases, Arg and Met exerted a different influence on the same parameter, as demonstrated by the results of the one-way ANOVA. According to Ghamari Monavvar et al. [33], arginine, as a precursor of polyamines and nitric oxide, stimulates protein synthesis and the proliferation and migration of intestinal cells, thereby improving intestinal morphometry. Nitric oxide also stimulates glucose uptake from the digesta, systemic immunity, and hormone secretion. Liu et al. [47] showed that in arginine deficiency, the enzyme nitric oxide synthase (EC 1.14.13.39) (physiologically responsible for nitric oxide production) can generate the production of free radicals with neurodegenerative effects. Notably, nitric oxide is derived from arginine; nitric oxide synthase itself is dependent on Ca^2+^/calmodulin, and its coenzyme is NADPH^+^ H^+^. The efficiency of this enzyme depends on an appropriate concentration of Ca^2+^ ions in the neuron, and excessive calcium inhibits the activity of nitric oxide synthase. Interestingly, research by Keith et al. [48] shows that amyloid-β can increase nitric oxide production by this enzyme and at the same time stimulate synthesis of the protein component of the enzyme. Zeng et al. [49] report that the neurotoxicity of amyloid-β worsens the dysfunction of the blood–brain barrier, increasing oxidative stress and inflammation of the nervous system, which can lead to the impairment of blood vessel function [49]. According to the vascular theory of amyloid plaque formation, impairment of the blood–brain barrier may allow blood proteins to penetrate the brain, including components of hemoglobin. Blood proteins generated from metabolic transformations and hemoglobin proteins in the brain tissue can both stimulate production of amyloid β [50].

According to Butterfield et al. [51], neurodegenerative changes induced by the presence of amyloid-β and free radicals involve oxidative damage to key enzymes taking part in glycolysis, the tricarboxylic acid cycle, and the biosynthesis of ATP. This type of damage to processes associated with biological oxidation in neurons adversely affects the metabolism of glucose, a key energy source for the brain, and then results in a characteristic decrease in cerebral glucose metabolism [50]. On the other hand, glucogenic amino acids such as arginine and methionine can be converted into one of the intermediate compounds of the Krebs cycle, which during successive reactions can be utilized for glucose synthesis. In addition, metabolism of arginine and methionine, similar to lysine metabolism, generates large amounts of ATP in the cell. Moreover, each molecule of the glucogenic amino acid donates one molecule of NADPH^+^ H^+^, which is used for the synthesis of fatty acids or cholesterol. A high concentration of cholesterol stimulates the production of amyloid-β from APP by activating β-secretase, which is much more efficient in a cholesterol-rich environment. It may be that a 110% proportion of Arg relative to Lys [5] is conducive to these conditions, resulting in an increased production of amyloid-β and a decrease in the level of LRP1. No such relationship was observed in the case of 110% Arg relative to the Lys level recommended by BUT [6]. As reported by Chen et al. [35] the neuroprotective action of Arg consists in inhibiting inflammatory processes, preventing oxidative stress, and improving cerebral circulation thanks to nitric oxide, for which Arg is a substrate. Chen et al. [35] argue that the neuroprotective effects of Arg can be enhanced by stimulating the metabolism of this amino acid by adding Met to a poultry diet. According to this author, the neuroprotection is possible thanks to the regulation of the transcription factor HIF-1α/LDHA, which is formed from arginine. The molecular mechanism of arginine-mediated neuroprotection is by suppression of the HIF-1α/LDHA signaling pathway during hypoxia, resulting in an inhibition of the inflammatory response. Inhibition of the inflammatory neuron response by attenuating HIF-1α/LDHA signaling exerts neuroprotective effects and reduces the occurrence of degenerative changes. Such reactions also regulate glycolysis; by improving this process in neurons, the inflammatory reaction is inhibited. In turn, arginine deficiencies inhibit glycolysis and redirects the cell’s metabolism, which stimulates the formation of toxic protein deposits, i.e., amyloid and the Tau protein deposits [52]. According to Kremer et al. [53] arginine may influence the synergistic effect between the urea cycle and glycolysis. Arginine is hydrolyzed by the enzyme arginase, leading to the production of urea and ornithine. According to Całyniuk et al. [24] urea production increases with a rise in the Arg content of bird diets, but excess Arg has no significant effect on Lys metabolism, whereas excess Lys strongly antagonizes the metabolism of Arg.

Neurodegenerative changes in liver cells are also associated with disturbances of mitochondrial function. On the one hand, toxic substances for neurons are produced, and on the other hand, processes that are associated with energy acquisition for neurons are disturbed [54]. In our study, a beneficial reduction in amyloid-β in the brain tissue of turkeys was obtained in Experiment 2 by increasing the Met level from 30 to 45% of the Lys level while maintaining the 1:1 ratio for Lys and Arg [6]. It is likely that this is the optimum dose for protecting brain and liver tissue, because it does not initiate neurodegenerative changes. Among Arg levels proposed in BUT [6] guidelines for turkey diets, 90 or 110% of the Lys level in combination with 30 or 45% Met, can be considered optimal in terms of neuroprotective effects. A study by Tapia-Rojas et al. [7] in mice demonstrated that a diet with too much Met can increase levels of amyloid-β and phosphorylated Tau protein, which are indicative of neurodegenerative changes. According to Zhang et al. [55], an increase in amyloid-β levels may be due to intensification of the methionine cycle reaction, which causes an increase in the activity of γ-secretase and APP cleavage, resulting in an increase in amyloid-β protein in the brain tissue [54].

Unfortunately, as the highest GAChE level was noted in the liver of turkeys from the treatment using 100% Arg and 45% Met (relative to the Lys level recommended by BUT [6], it seems likely that large amounts of amyloid-β were involved in the formation of these toxic complexes, which consist of amyloid-β, Tau protein, and inactive AChE. According to Reitz and Mayeux [56] and Kamal at al. [57], the deposition of amyloid-β in the central nervous system takes place in the neuronal axonal membranes, disturbing homeostasis in nerve cells and leading to abnormal APP metabolism and the overproduction of amyloid-β peptides [58]. Metabolic disturbances in liver hepatocytes and neurons also cause the glycosylation of acetylcholinesterase, i.e., complexes of acetylcholinesterase with amyloid-β and Tau protein. These toxic complexes, called glycosylated acetylcholinesterase (GAChE), stimulate apoptosis of both hepatocytes and neurons. In the case of a change in the level of Arg relative to the Lys level recommended by BUT [6], the variant with a 110% share of Arg relative to Lys seems to be much more favorable, because it reduces the concentration of GAChE and methylation changes in the brain tissue. The 90% share of Arg relative to Lys [6] caused a very promising increase in the LRP1 level in the brain tissue. Another cause of metabolic disturbances in the neurons is the formation of complexes of AChE and Tau protein, known as GAChE. According to Lesné et al. [59], GAChE causes an activation of glutamate receptor NMDA, inducing the mass influx of Ca^2+^ and a non-physiological elevation of the intracellular concentration of this ion in the nerve cell. This leads to a reduction in the rate of oxidative phosphorylation and the ATP level in the neuron [25,26,27] disturbing the metabolism of this cell. The most neurotoxic effects of GAChE include the activation of the glutaminergic NMDA receptor (N-methyl-D-aspartate receptor), the opening of calcium channels, activation of calcium/calmodulin-dependent kinase II, and the activation of caspases [25,26,27]. Activation of glutamate NMDA receptor induces a mass influx of Ca^2+^ and a non-physiological elevation of the intracellular concentration of this ion in the cell, which leads to a decrease in the rate of oxidative phosphorylation and the ATP level. Disruption of cellular respiration is conducive to the opening of mitochondrial mega channels, which can lead to the release of cytochrome C from the intermembrane space and activation of caspase 3. The disruption of cellular homeostasis leads to apoptotic cell death [25,26,27]. Another result of the neurodegenerative effects of both amyloid-β itself and GAChE is the generation of reactive oxygen species, especially in the presence of Cu^2+^ and Zn^2+^ ions. This results in oxidative stress and a decrease in ATP production in the neurons, which in combination with disturbances of Ca^2+^ metabolism induces a number of pathological changes leading to the death of neurons [60].

In both Experiments 1 and 2, the varied levels of Met relative to Lys had no negative effect on indicators of neurodegeneration in the brain. Therefore, a Met level amounting to 30 or 45% of the lysine level can be considered to be beneficial, causing no neurodegenerative changes. In the present study, the level of Tau protein in the brain of turkeys decreased when the Met level in the diet was increased to 45% of the Lys level and Arg content was reduced to 90% of the Lys level (recommended by BUT) [6]. According to Lesné et al. [59], Met reduces the activity of Tau kinase, thereby promoting the dissociation of Tau protein from microtubules, which results in the formation of neurofibrillary tangles, manifesting the onset of neurodegenerative changes. Kinase and phosphatase are responsible for the phosphorylation of Tau protein, and an imbalance between these enzymes leads to the hyperphosphorylation of Tau protein. Tau protein may also undergo other post-translation modifications, including acetylation, which magnifies its role in the development of neurodegenerative changes [61]. The results of our study indicate that increasing the proportion of Arg to 110% of the Lys level proposed by BUT [6] causes an unfavorable decrease in the AChE level, but this is accompanied by a highly favorable decrease in the concentration of GAChE molecules and the percentage of DNA methylation in the brain tissue. Reducing Arg to 90% of BUT [6] guidelines caused a highly favorable increase in the LRP1 level in the brain tissue.

According to Sharma [62], the declining level of AChE in neurodegenerative diseases leads to the accumulation of large amounts of acetylcholine and to overstimulation of the cholinergic system. In our study, the higher AChE level in the liver of turkeys receiving an increased addition of Arg to 110% relative to Lys (in comparison to BUT guidelines) [6] should be considered to be highly favorable. In contrast, the consequences of using Arg in the amount of 90% of the Lys level (NRC) [5] were clearly unfavorable, as the level of Tau protein in the brain increased. According to Méndez et al., Elufioye et al., and Ikonomovic et al. [63,64,65], a decrease in AChE activity results in mitochondrial dysfunction and inflammation in brain cells. In addition, reduced AChE activity inhibits certain complexes of the respiratory chain, leading to an increased production of toxic free radicals, especially hydroxyl radical and superoxide ions, inducing neuroinflammatory changes [66]. According to Day and Greenfield [25,26,27], a peptide can detach from the C-terminal of the AChE molecule and attach to a glycosylated fragment of the amyloid-β precursor or Tau protein [34], forming a molecule of glycosylated acetylcholinesterase (GAChE). A decrease in the level of AChE, the main enzyme of cholinergic synapses in the brain and neuromuscular connections, may therefore be an early symptom of neuropathological changes [67,68,69]. According to Méndez et al. [63], the most severe changes of this type are observed in the cerebral cortex and hippocampus and are accompanied by a significant increase in the cholesterol level in these tissues [68]. The high level of Lys and Met used in Experiment 1 (110 and 45% in relation to Lys), in relation to the nutritional recommendations of the NRC, according to Ognik et al. [22], can stimulate the synthesis of cholesterol, while a high level of Arg in the diet may partially counteract such reactions. Due to the fact that high cholesterol according to the NRC [5] clearly stimulates the production of amyloid-β, only its supplementation in accordance with the BUT [6] requirements can be considered neuroprotective. An additional premise supporting this fact may be the ability of Arg to stimulate the urea cycle. The product of this process, i.e., urea, has the ability to change the conformational structure of amyloid-β, which causes the unfolding of the structure of this molecule [70]. Xiang et al. [70] suggest that the level of this neurotransmitter may be decreased by acetylcholine-binding amyloid-β. In Experiment 2, increasing the Met level to 45% and the Arg level to 110% of the Lys level recommended by BUT [6] had highly diverse effects. It caused an unfavorable increase in the GAChE level accompanied by a very promising decrease in the level of amyloid-β itself in the liver tissue of the turkeys. Unfortunately, because GAChE is a complex of amyloid-β and Tau protein with molecules of the enzyme AChE, this result should probably be considered unfavorable, as this type of complex causes the blockage of active AChE, a valuable enzyme for normal neuron function.

To verify the hypotheses put forward in this paper, further research should include other useful indicators of neurodegenerative changes, especially levels of APP, crucial enzymes (calcium/calmodulin-dependent protein kinase type II (CAMK2), calcium/calmodulin-dependent protein kinase type IV (CAMK4) and glucose 6 phosphate dehydrogenase), calcium, vitamins, especially B6 and B12, thyroid hormones, and biomarkers of inflammation and oxidative stress (cytokines and isoprostanes). Microscopic examination may prove useful as well (observation of plaques formed of Tau protein and clusters of amyloid β).

## 5. Conclusions

It was established that in the case of both a low (NRC) [5] and a high (BUT) [6] level of Lys in the diet of turkeys, the Arg level can be reduced to 90% of the Lys level and Met to 30% of the Lys level, because this does not cause neurodegenerative changes in turkeys. Unfavorable neurodegenerative changes may appear if the Arg level is increased from 100 to 110% of the Lys level recommended by the NRC [5].

Among the tested proportions of Lys and Arg in the diet of meat chickens, the safest variant is the higher level of lysine recommended by BUT [6] and an increase in the Arg level to 110% of that Lys level. In our opinion, these proportions of amino acids in the diet, among those tested, are the most effective at limiting neurodegenerative changes.

## Figures and Tables

**Table 1:** Ingredient composition and nutrient content of basal diets (g/kg, as-fed basis) fed to turkeys at 1–4, 5–8, 9–12, and 13–16 weeks of age, Experiment 1 ^1^.

Item	Feeding Period, Weeks			
	1–4	5–8	9–12	13–16
Ingredients				
Wheat	463.9	486.7	537.4	656.3
Maize	100.0	100.0	100.0	100.0
Soybean meal	250.5	232.7	187.3	79.1
Rapeseed meal	30.0	50.0	71.8	70.0
Potato protein	55.2	30.1	-	-
Soybean oil	2.0	23.2	35.3	32.2
Maize gluten meal	55.0	35.0	35.0	35.0
Sodium bicarbonate	2.0	2.0	2.0	2.0
Sodium chloride	1.5	1.6	1.6	1.4
Limestone	22.0	18.6	16.4	13.8
Monocalcium phosphate	14.6	12.9	9.0	5.0
L-Threonine	-	0.7	0.7	1.7
Choline chloride	1.0	1.0	1.0	1.0
Vitamin-mineral premix ^2^	2.5	2.5	2.5	2.5
Titanium oxide	-	3.0	-	-
Calculated nutrient content				
Metabolizable energy, kcal/kg	2820	2900	3000	3100
Crude protein	26.5	23.5	20.5	17.0
Arginine total ^3^	14.4	13.5	11.7	8.9
Lysine total ^3^	12.8	11.2	8.9	6.4
Methionine total ^3^	4.5	3.9	3.4	2.9
Methionine + Cysteine total	9.2	8.2	8.0	7.5
Threonine total	10.2	9.5	7.4	6.5
Calcium	12.5	11.0	9.5	7.5
Available phosphorus	6.5	5.5	4.7	3.8

^1^ Source: This table was published in Poultry Science [2]. ^2^ Provided per kg diet (feeding periods: weeks 0–4, 5–8, 9–12 and 13–16): mg: retinol 3.78, 3.38, 2.88 and 2.52; cholecalciferol 0.13, 0.12, 0.10 and 0.09; α-tocopheryl acetate 100, 90, 80 and 70; vit. K_3_ 5.8, 5.6, 4.8 and 4.2; thiamine 5.4, 4.7, 4.0 and 3.5; riboflavin 8.4, 7.5, 6.4 and 5.6; pyridoxine 6.4, 5.6, 4.8 and 4.2; cobalamin 0.032, 0.028, 0.024 and 0.021′ biotin 0.32, 0.28, 0.24 and 0.21; pantothenic acid 28, 24, 20 and 18; nicotinic acid 84, 75, 64 and 56; folic acid 3.2, 2.8, 2.4 and 2.1; Fe 64, 60, 56, 48 and 42; Mn 120, 112, 96 and 84; Zn 110, 103, 88 and 77; Cu 23, 19, 16 and 14; I 3.2, 2.8, 2.4 and 2.1; Se 0.30, 0.28, 0.24 and 0.21, respectively. ^3^ Actual levels of supplementary Lys, Arg, and Met in experimental diets were obtained by adding supplementary L-Lys HCl, L-Arg HCl and DL-Met to the basal feed. L-Lys HCl was added to the basal diet to obtain 1.60, 1.50, 1.30, and 1.00 g of Lys per 100 g of feed in four successive feeding periods, according to the nutrient requirements of turkeys [5]. L-Arg HCl was added to the basal diet to obtain 90, 100, and 110% Arg relative to the content of dietary Lys. DL-Met was added to obtain 30 and 45% Met relative to the content of dietary Lys. Lys—lysine, Arg—arginine, Met—methionine.

**Table 2:** Amino acid content (g/kg) of basal diets of turkeys, Experiment 1 ^1^.

Item	Feeding Period, Weeks			
	1–4	5–8	9–12	13–16
Crude protein	270.8	246.3	209.3	177.0
Alanine	13.27	11.04	9.87	9.16
Arginine	14.81	13.70	11.93	9.16
Aspartic acid	25.40	21.22	17.88	11.66
Cysteine	4.62	4.09	3.77	3.34
Glutamic acid	53.73	46.06	41.84	37.12
Glycine	11.42	9.72	8.69	6.69
Histidine	6.49	5.67	5.18	4.44
Isoleucine	11.93	9.83	8.68	6.23
Leucine	24.24	19.76	17.33	13.25
Lysine	12.96	11.89	9.62	6.22
Mathionine	4.56	3.94	3.36	2.47
Methionine + Cysteine	9.18	8.03	7.13	5.81
Phenyloalanine	14.56	11.91	10.35	7.81
Proline	18.15	15.89	14.92	14.60
Serine	13.66	11.47	10.13	7.72
Threonine	10.61	9.09	7.68	6.76
Tyrosine	8.70	8.19	7.51	5.41
Valine	13.50	11.34	9.84	7.34

^1^ Source: This table was published in Poultry Science [2].

**Table 3:** Ingredient composition and nutrient content of basal diets (g/kg, as-fed basis) fed to turkeys at 1–4, 5–8, 9–12, and 13–16 weeks of age, Experiment 2 ^1^.

Item	Feeding Period, Weeks			
	1–4	5–8	9–12	13–16
Ingredients				
Wheat	439.8	471.2	519.9	617.1
Maize	100.0	100.0	100.0	100.0
Soybean meal, 48% CP	287.7	265.4	238.5	152.4
Rapeseed meal	30.0	30.0	30.0	30.0
Potato protein	50.0	29.6	-	-
Soybean oil	9.5	28.5	47.8	42.2
Maize gluten meal	35.0	30.0	30.0	30.0
Sodium bicarbonate	2.0	2.0	2.0	2.0
Sodium chloride	1.5	1.6	1.6	1.2
Limestone	20.7	18.7	16.4	14.5
Monocalcium phosphate	19.4	15.5	9.6	6.5
L-Threonine	0.9	1.0	0.7	0.6
Choline chloride	1.0	1.0	1.0	1.0
Vitamin-mineral premix ^2^	2.5	2.5	2.5	2.5
Titanium oxide	-	3.0	-	-
Calculated nutrient content				
Metabolizable energy, kcal/kg	2820	2950	3000	3150
Crude protein	27.0	24.5	21.5	18.5
Arginine total ^3^	1.58	1.44	1.27	1.04
Lysine total ^3^	1.36	1.19	0.97	0.76
Methionine total ^3^	0.44	0.39	0.34	0.30
Methionine + Cysteine total	0.91	0.83	0.74	0.67
Threonine total	1.02	1.01	0.83	0.70
Calcium	1.30	1.15	0.95	0.80
Available phosphorus	0.70	0.60	0.47	0.40

^1^ Source: This table was published in Poultry Science [3]. ^2^ Provided per kg diet (feeding periods: weeks 0–4, 5–8, 9–12 and 13–16): mg: retinol 3.78, 3.38, 2.88, and 2.52; cholecalciferol 0.13, 0.12, 0.10, and 0.09; α-tocopherol acetate 100, 90, 80, and 70; vit. K_3_ 5.8, 5.6, 4.8, and 4.2; thiamine 5.4, 4.7, 4.0, and 3.5; riboflavin 8.4, 7.5, 6.4, and 5.6; pyridoxine 6.4, 5.6, 4.8, and 4.2; cobalamin 0.032, 0.028, 0.024, and 0.021; biotin 0.32, 0.28, 0.24, and 0.21; pantothenic acid 28, 24, 20, and 18; nicotinic acid 84, 75, 64, and 56; folic acid 3.2, 2.8, 2.4, and 2.1; Fe 64, 60, 56, 48, and 42; Mn 120, 112, 96, and 84; Zn 110, 103, 88, and 77; Cu 23, 19, 16, and 14; I 3.2, 2.8, 2.4, and 2.1; Se 0.30, 0.28, 0.24, and 0.21, respectively. ^3^ Actual levels of supplementary Lys, Arg, and Met in experimental diets were obtained by adding supplementary L-Lys HCl, L-Arg HCl, and DL-Met to the basal feed. L-Lys HCl was added to the basal diet to obtain 1.60, 1.50, 1.30, and 1.00 g of Lys per 100 g of feed in four successive feeding periods, according to the nutrient requirements of turkeys [6]. L-Arg HCl was added to the basal diet to obtain 90, 100, and 110% Arg relative to the content of dietary Lys. DL-Met was added to obtain 30 and 45% Met relative to the content of dietary Lys. Lys—lysine, Arg—arginine, Met—methionine.

**Table 4:** Amino acid content (g/kg) of basal diets of turkeys, Experiment 2 ^1^.

Item	Feeding Period, Weeks			
	1–4	5–8	9–12	13–16
CP	265.7	248.0	221.0	195.50
Alanine	11.9	12.3	10.0	8.0
Arginine	15.6	14.2	13.7	11.1
Aspartic acid	24.1	23.4	17.7	14.4
Cysteine	4.2	3.9	2.9	2.6
Glutamic acid	45.0	51.0	43.8	43.0
Glycine	10.2	11.3	9.0	8.3
Histidine	6.5	6.7	5.4	5.2
Isoleucine	10.5	10.6	8.6	7.7
Leucine	20.4	21.4	17.9	15.7
Lysine	13.7	12.1	10.2	8.1
Methionine	4.2	3.9	2.9	2.6
Methionine + Cysteine	8.4	7.9	6.5	5.9
Phenylalanine	11.6	12.9	10.9	9.0
Proline	13.8	19.0	14.4	15.7
Serine	11.7	13.0	9.8	8.3
Threonine	10.6	11.1	8.4	7.3
Tyrosine	9.6	10.1	7.5	6.6
Valine	11.7	12.1	10.5	9.0

^1^ Source: This table was published in Poultry Science [3].

**Table 5:** Indicators of neurodegenerative changes in the brain of turkeys, Experiment 1.

Treatment ^1^	AChEng/mL	GAChEng/mL	Amyloid-β pg/mL	LRP 1 pg/mL	Taung/mL	% Methylation
Arg_90_Met_30_	23.21	0.323	128.89	210.85	34.56 ^ab^	61.37
Arg_90_Met_45_	20.91	0.337	145.82	254.25	34.93 ^ab^	69.41
Arg_100_Met_30_	26.52	0.253	137.29	212.86	31.67 ^b^	65.45
Arg_100_Met_45_	26.72	0.29	140.38	265.25	35.92 ^a^	67.22
Arg_110_Met_30_	31.54	0.261	174.86	214.66	31.65 ^b^	66.44
Arg_110_Met_45_	26.51	0.266	190.02	219.29	33.39 ^ab^	69.24
SEM	0.015	0.004	0.145	0.526	0.047	0.022
Arg level, %						
90	22.06 ^c^	0.330	137.36 ^b^	232.55 ^a^	34.75 ^b^	65.39
100	26.62 ^b^	0.272	138.84 ^b^	239.01 ^a^	33.79 ^b^	66.34
110	30.21 ^a^	0.296	158.56 ^a^	205.14 ^b^	48.08 ^a^	67.84
Met level, %						
30	27.85	0.27	161.03	218.33	32.75	64.42
45	26.18	0.286	154.04	227.93	33.55	68.62
*p*-value						
Arg	0.019	0.129	0.032	0.035	0.002	0.147
Met	0.062	0.235	0.075	0.127	0.225	0.221
Arg × Met	0.057	0.092	0.235	0.357	0.051	0.187

^abc^ values in same column with no common superscript denote a significant difference (*p* ≤ 0.05). Abbreviations: Arg, arginine; Lys, lysine; Met, methionine; SEM, standard error of mean; AChE, acetylcholinesterase; GAChE, glycosylated acetylcholinesterase; LRP 1, low-density lipoprotein receptor-related protein 1; Tau, Tau protein. ^1^ Treatment: Arg_90_Met_30_ received 90% Arg and 30% Met relative to the content of dietary Lys; Arg_90_Met_45_ received 90% Arg and 45% Met relative to the content of dietary Lys; Arg_100_Met_30_ received 100% Arg and 30% Met relative to the content of dietary Lys; Arg_100_Met_45_ received 100% Arg and 45% Met relative to the content of dietary Lys; Arg_110_Met_30_ received 110% Arg and 30% Met relative to the content of dietary Lys; Arg_110_Met_45_ received 110% Arg level and 45% Met level relative to the content of dietary Lys.

**Table 6:** Indicators of neurodegenerative changes in the brain of turkeys, Experiment 2.

Treatment ^1^	AChEng/mL	GAChEng/mL	Amyloid-β pg/mL	LRP 1 pg/mL	Taung/mL	% Methylation
Arg_90_Met_30_	32.83	0.279	164.92 ^a^	250.62	104.11 ^a^	75.54 ^ab^
Arg_90_Met_45_	27.70	0.316	176.95 ^a^	229.86	37.19 ^b^	69.36 ^b^
Arg_100_Met_30_	27.82	0.262	168.38 ^a^	196.14	33.42 ^b^	65.16 ^b^
Arg_100_Met_45_	28.80	0.329	140.72 ^b^	186.03	32.38 ^b^	86.88 ^ab^
Arg_110_Met_30_	30.96	0.335	133.53 ^b^	177.65	35.59 ^b^	105.61 ^a^
Arg_110_Met_45_	34.98	0.253	158.46 ^ab^	170.46	38.42 ^b^	99.33 ^a^
SEM	0.022	0.012	0.245	0.078	0.069	0.124
Arg level, %						
90	30.27 ^a^	0.298 ^a^	170.94 ^a^	240.24 ^a^	70.65 ^a^	72.45 ^a^
100	28.31 ^a^	0.296 ^a^	154.55 ^b^	190.09 ^b^	32.90 ^b^	76.02 ^a^
110	17.08 ^c^	0.156 ^c^	151.12 ^b^	201.96 ^b^	36.25 ^b^	17.08 ^b^
Met level, %						
30	30.87	0.293	153.13	195.39	46.87	86.94
45	30.57	0.295	156.58	200.88	46.85	84.12
*p*-value						
Arg	≤0.001	≤0.001	0.032	0.047	0.033	≤0.001
Met	0.235	0.182	0.075	0.069	0.082	0.066
Arg × Met	0.125	0.236	0.042	0.075	0.035	0.079

^abc^ values in same column with no common superscript denote a significant difference (*p* ≤ 0.05). Abbreviations: Arg, arginine; Lys, lysine; Met, methionine; SEM, standard error of mean; AChE, acetylcholinesterase; GAChE, glycosylated acetylcholinesterase; LRP 1, low-density lipoprotein receptor-related protein 1; Tau, Tau protein. ^1^ Treatment: Arg_90_Met_30_ received 90% Arg and 30% Met relative to the content of dietary Lys; Arg_90_Met_45_ received 90% Arg and 45% Met relative to the content of dietary Lys; Arg_100_Met_30_ received 100% Arg and 30% Met relative to the content of dietary Lys; Arg_100_Met_45_ received 100% Arg and 45% Met relative to the content of dietary Lys; Arg_110_Met_30_ received 110% Arg and 30% Met relative to the content of dietary Lys; Arg_110_Met_45_ received 110% Arg level and 45% Met level relative to the content of dietary Lys.

**Table 7:** Indicators of neurodegenerative changes in the liver of turkeys, Experiment 2.

Treatment ^1^	AChEng/mL	GAChEng/mL	Amyloid-β pg/mL	LRP 1 pg/mL	Taung/mL	% Methylation
Arg_90_Met_30_	20.61	0.183 ^b^	219.54 ^a^	185.51	35.29	75.85
Arg_90_Met_45_	17.82	0.134 ^b^	195.24 ^a^	209.19	37.41	69.77
Arg_100_Met_30_	15.76	0.103 ^c^	75.22 ^c^	181.07	36.98	65.71
Arg_100_Met_45_	14.99	0.090 ^c^	79.79 ^c^	219.39	37.36	81.01
Arg_110_Met_30_	14.75	0.075 ^c^	185.59 ^c^	203.4	33.71	75.78
Arg_110_Met_45_	18.12	0.406 ^a^	168.71 ^b^	219.54	35.69	79.47
SEM	0.03	0.008	0.145	0.167	0.047	0.095
Arg level, %						
90	19.22 ^a^	0.159 ^a^	207.39 ^a^	197.35	36.35	72.81
100	15.38 ^b^	0.097 ^b^	77.51 ^c^	200.23	37.17	73.36
110	17.08 ^a^	0.156 ^a^	151.12 ^b^	201.96	36.25	77.63
Met level, %						
30	16.64	0.156	151.89	199.30	35.54	72.45
45	16.96	0.164	153.71	202.49	35.99	76.75
*p*-value						
Arg	0.022	0.018	≤0.001	0.682	0.799	0.574
Met	0.326	0.095	0.228	0.072	0.083	0.087
Arg × Met	0.093	0.008	0.025	0.374	0.189	0.121

^abc^ values in same column with no common superscript denote a significant difference (*p* ≤ 0.05). Abbreviations: Arg, arginine; Lys, lysine; Met, methionine; SEM, standard error of mean; AChE, acetylcholinesterase; GAChE, glycosylated acetylcholinesterase; LRP 1, low-density lipoprotein receptor-related protein 1; Tau, Tau protein. ^1^ Treatment: Arg_90_Met_30_ received 90% Arg and 30% Met relative to the content of dietary Lys; Arg_90_Met_45_ received 90% Arg and 45% Met relative to the content of dietary Lys; Arg_100_Met_30_ received 100% Arg and 30% Met relative to the content of dietary Lys; Arg_100_Met_45_ received 100% Arg and 45% Met relative to the content of dietary Lys; Arg_110_Met_30_ received 110% Arg and 30% Met relative to the content of dietary Lys; Arg_110_Met_45_ received 110% Arg level and 45% Met level relative to the content of dietary Lys.

## Data Availability

The data presented in this study are available in the article.

## References

[1] Fouad A.M., El-Senousey H.K., Yang X.J., Yao J.H. (2012). Role of dietary L-arginine in poultry production. Int. J. Poult. Sci..

[2] Jankowski J., Mikulski D., Mikulska M., Ognik K., Całyniuk Z., Mróz E., Zduńczyk Z. (2019). The effect of different dietary ratios of arginine, methionine, and lysine on the performance, carcass traits, and immune status of turkeys. Poult. Sci..

[3] Jankowski J., Ognik K., Konieczka P., Mikulski D. (2020). Effects of different levels of arginine and methionine in a high-lysine diet on the immune status, performance, and carcass traits of turkeys. Poult. Sci..

[4] Balnave D., Brake J. (2002). Re-evaluation of the classical dietary arginine: Lysine interaction for modern poultry diets: A review. Worlds Poult. Sci. J..

[5] NRC: Research Council National (1994). Nutrient Requirements of Poultry.

[6] British United Turkeys (BUT), Aviagen Turkeys Management Guidelines for Raising Commercial Turkeys. https://www.aviagenturkeys.com/media/183481/aviagencommercialguide.pdf.

[7] Tapia-Rojas C., Lindsay C.B., Montecinos-Oliva C., Arrazola M.S., Retamales R.M., Bunout D., Hirsch S., Inestrosa N.C. (2015). Is L-methionine a trigger factor for Alzheimer’s-like neurodegeneration? Changes in Aβ oligomers, tau phosphorylation, synaptic proteins, Wnt signaling and behavioral impairment in wild-type mice. Mol. Neurodegener..

[8] Morales I., Guzmán-Martínez L., Cerda-Troncoso C., Farías G.A., Maccioni R.B. (2014). Neuroinflammation in the pathogenesis of Alzheimer’s disease. A rational framework for the search of novel therapeutic approaches. Front. Cell Neurosci..

[9] Tu R., Grover H.M., Kotra L.P. (2016). Peptidyl Arginine Deiminases and Neurodegenerative Diseases. Curr. Med. Chem..

[10] Tóthová B., Kovalska M., Kalenská D., Tomascova A., Lehotský J. (2017). Effect of methionine-induced hyperhomocysteinemia on neurodegeneration in experimental conditions. Act. Nerv. Super. Rediviva.

[11] Toue S., Kodama R., Amao M., Kawamata Y., Kimura T., Sakai R. (2006). Screening of toxicity biomarkers for methionine excess in rats. J. Nutr..

[12] Koladiya R.U., Jaggi A.S., Singh N., Sharma B.K. (2008). Ameliorative role of Atorvastatin and Pitavastatin in L-Methionine induced vascular dementia in rats. BMC Pharm..

[13] Goren J.L., Stoll A.L., Damico K.E., Sarmiento I.A., Cohen B.M. (2004). Bioavailability and lack of toxicity of S-adenosyl-L-methionine (SAMe) in humans. Pharmacotherapy.

[14] Ringman J.M., Coppola G. (2013). New genes and new insights from old genes: Update on Alzheimer disease. Contin. Lifelong Learn. Neurol..

[15] Stadtman E.R., Van Remmen H., Richardson A., Wehr N.B., Levine R.L. (2005). Methionine oxidation and aging. Biochim. Biophys. Acta.

[16] Vuaden F.C., Savio L.E., Piato A.L., Pereira T.C., Vianna M.R., Bogo M.R., Bonan C.D., Wyse A.T. (2012). Long-term methionine exposure induces memory impairment on inhibitory avoidance task and alters acetylcholinesterase activity and expression in zebrafish (Danio rerio). Neurochem. Res..

[17] Cudic P., Joshi N., Sagher D., Williams B.T., Stawikowski M.J., Weissbach H. (2015). Identification of activators of methionine sulfoxide reductases A and B. Biochem. Biophys. Res. Commun..

[18] Olney J.W., Zorumski C., Price M.T., Labruyere J. (1990). L-cysteine, a bicarbonate-sensitive endogenous excitotoxin. Science.

[19] Khajali F., Wideman R. (2010). Dietary arginine: Metabolic, environmental, immunological and physiological interrelationships. Worlds Poult. Sci. J..

[20] Rajagopal S., Joginapally V.R., Srinivasan T.S., Ramanathan V., Essa M.M., Memon M.A. (2014). Beneficial Effects of Dietary Amino Acids on Brain Health.

[21] Oso A.O., Williams G.A., Oluwatosin O.O., Bamgbose A.M., Adebayo A.O., Olowofeso O.V., Pirgozliev A.A., Adegbenjo S.O., Osho J.O., Alabi F. (2017). Effect of dietary supplementation with arginine on hematological indices, serum chemistry, carcass yield, gut microflora, and lymphoid organs of growing turkeys. Livest. Sci..

[22] Bouchereau J., Schiff M. (2020). Inherited Disorders of Lysine Metabolism: A Review. J. Nutr..

[23] Ognik K., Całyniuk Z., Mikulski D., Stępniowska A., Konieczka P., Jankowski J. (2021). The effect of different dietary ratios of lysine, arginine and methionine on biochemical parameters and hormone secretion in turkeys. J. Anim. Physiol. Anim. Nutr..

[24] Całyniuk Z., Cholewińska E., Konieczka P., Ognik K., Mikulski D., Jankowski J. (2021). The effect of the application of diets with varied proportions of arginine and lysine on biochemical and antioxidant status in turkeys. Ann. Anim. Sci..

[25] Day T., Greenfield S.A. (2002). A non-cholinergic, trophic action of acetylcholinesterase on hippocampal neurons in vitro: Molecular mechanisms. Neuroscience.

[26] Day T., Greenfield S.A. (2003). Bioactivity of a peptide derived from acetylcholinesterase in hippocampal organotypic cultures. Exp. Brain Res..

[27] Day T., Greenfield S.A. (2003). A peptide derived from acetylcholinesterase induces neuronal cell death: Characterization of possible mechanisms. Exp. Brain Res..

[28] Hasani S.A., Mayeli M., Salehi M.A., Barzegar Parizi R. (2021). A Systematic Review of the Association between Amyloid-β and τ Pathology with Functional Connectivity Alterations in the Alzheimer Dementia Spectrum Utilizing PET Scan and rsfMRI. Dement. Geriatr. Cogn. Disord. Extra..

[29] Rowe E.M., Xing V., Biggar K.K. (2019). Lysine methylation: Implications in neurodegenerative disease. Brain Res.

[30] Jankowski J., Ognik K., Całyniuk Z., Stępniowska A., Konieczka P., Mikulski D. (2021). The effect of different dietary ratios of lysine, arginine and methionine on protein nitration and oxidation reactions in turkey tissues and DNA. Animal.

[31] Tufarelli V., Mehrzad-Gilmakek H., Booyeh M., Qotbi A., Seidavi A., Paz A.E., Laudadio V. (2020). Effect of different levels of L-carnitine and excess lysine-methionine on broiler performance, carcass characteristics, blood constituents, immunity and triiodothyronine hormone. Agriculture.

[32] Ghasemabad B. (2017). Effects of copper sulfate and arginine supplements on performance and carcass traits in broiler chickens fed with canola meal based diet. Iran. J. Appl. Anim. Sci..

[33] Ghamari Monavvar H., Moghaddam G., Ebrahimi M. (2020). A review on the effect of arginine on growth performance, meat quality, intestine morphology, and immune system of broiler chickens. Iran. J. Appl. Anim. Sci..

[34] Ghoreyshi S.M., Omri B., Chalghoumi R., Bouyeh M., Seidavi A.R., Dadashbeiki M., Lucarini M., Durrazo A., van den Hoven R., Santini A. (2019). Effects of Dietary Supplementation of L-Carnitine and Excess Lysine-Methionine on Growth Performance, Carcass Characteristics, and Immunity Markers of Broiler Chicken. Animals.

[35] Chen S.F., Pan M.X., Tang J.C. (2020). Arginine is neuroprotective through suppressing HIF-1α/LDHA-mediated inflammatory response after cerebral ischemia/reperfusion injury. Mol. Brain.

[36] Li Y., Li K., Wang X., Cui M., Ge P., Zhang J., Qiu F., Zhong C. (2020). Conformable self-assembling amyloid protein coatings with genetically programmable functionality. Sci. Adv..

[37] Ghiso J., Tagliavini F., Frederik Timmers W., Frangione B. (2002). Alzheimer’s disease amyloid precursor protein is present in senile plaques and cerebrospinal fluid: Immunohistochemical and biochemical characterization. Biochem. Biophys. Res. Commun..

[38] Zlokovic B.V., Frangione B. (2003). Transport-clearance hypothesis for Alzheimer’s disease and potential therapeutic implications. Landes Biosci..

[39] Madra M., Sturley S.L. (2010). Niemann-Pick type C pathogenesis and treatment: From statins to sugars. Clin. Lipidol..

[40] Block R.C., Dorsey E.R., Beck C.A., Brenna J.T., Shoulson I. (2010). Altered cholesterol and fatty acid metabolism in Huntington disease. J. Clin. Lipidol..

[41] Di Paolo G., Kim T.W. (2011). Linking lipids to Alzheimer’s disease: Cholesterol and beyond. Nat. Rev. Neurosci..

[42] Wang Q., Yan J., Chen X., Li J., Yang Y., Weng J., Deng C., Yenari M.A. (2011). Statins: Multiple neuroprotective mechanisms in neurodegenerative diseases. Exp. Neurol..

[43] Estrada L.D., Ahumada P., Cabrera D., Arab J.P. (2019). Liver Dysfunction as a Novel Player in Alzheimer’s Progression: Looking Outside the Brain. Front. Aging Neurosci..

[44] Underhill S.M., Amara S.G. (2021). Acetylcholine Receptor Stimulation Activates Protein Kinase C Mediated Internalization of the Dopamine Transporter. Front. Cell Neurosci..

[45] Nikolakopoulou A.M., Wang Y., Ma Q., Sagare A.P., Montagne A., Huuskonen M.T., Sanket V., Kisler R.K., Dai Z., Körbelin J. (2021). Endothelial LRP1 protects against neurodegeneration by blocking cyclophilin A. J. Exp. Med..

[46] Oka M., Fujisaki N., Maruko-Otake A. (2017). Ca^2+^/calmodulin-dependent protein kinase II promotes neurodegeneration caused by tau phosphorylated at Ser262/356 in a transgenic Drosophila model of tauopathy. J. Biochem..

[47] Liu C., Liang M.C., Soong T.W. (2019). Nitric Oxide, Iron and Neurodegeneration. Front. Neurosci..

[48] Keith T., Akama L.J., Eldik V. (2020). β-Amyloid Stimulation of Inducible Nitric-oxide Synthase in Astrocytes Is Interleukin-1β- and Tumor Necrosis Factor-α (TNFα)-dependent, and Involves a TNFα Receptor-associated Factor- and NFκB-inducing Kinase-dependent Signaling Mechanism. J. Biol. Chem..

[49] Zeng P., Shi Y., Wang X.M., Lin L., Du Y.J., Tang N., Wang O., Fang Y.Y., Wang J.Z., Zhou X.W. (2019). Emodin Rescued Hyperhomocysteinemia-Induced Dementia and Alzheimer’s Disease-Like Features in Rats. Int. J. Neuropsychopharmacol..

[50] Wook K.J., Soo B.M., Dahyun Y., Ho L.Y., Yeon J.S., Kang K., Haejung J., Gijung J., Jun-Young L., Chul-Ho S. (2021). Blood Hemoglobin, in-vivo Alzheimer Pathologies, and Cognitive Impairment: A Cross-Sectional Study. Front. Aging Neurosci..

[51] Butterfield D.A., Halliwell B. (2019). Oxidative stress, dysfunctional glucose metabolism and Alzheimer disease. Nat. Rev. Neurosci..

[52] Baik S.H., Kang S., Lee W., Choi H., Chung S., Kim J.I., Mook-Jung I. (2019). A Breakdown in Metabolic Reprogramming Causes Microglia Dysfunction in Alzheimer’s Disease. Cell Metab..

[53] Kremer J.C., Prudner B.C., Lange S.E.S., Bean G.R., Schultze M.B., Brashears C.B., Van Tine B.A. (2017). Arginine deprivation inhibits the Warburg effect and upregulates glutamine anaplerosis and serine biosynthesis in ASS1-deficient cancers. Cell Rep..

[54] Liu Y.H., Wang Y.R., Xiang Y., Zhou H.D., Giunta B., Mañucat-Tan N.B. (2015). Clearance of amyloid-β in Alzheimer’s disease: Shifting the action site from center to periphery. Mol. Neurobiol..

[55] Zhang C., Wei W., Liu Y., Peng J., Tian Q., Liu G. (2009). Hyperhomocysteinemia increases beta-amyloid by enhancing expression of gamma-secretase and phosphorylation of amyloid precursor protein in rat brain. Am. J. Pathol..

[56] Reitz C., Mayeux R. (2014). Alzheimer Disease: Epidemiology, Diagnostic Criteria, Risk Factors and Biomarkers. Biochem. Pharm..

[57] Kamal A., Almenar-Queralt A., LeBlanc J.F., Roberts E.A., Goldstein L.S. (2001). Kinesin-mediated axonal transport of a membrane compartment containing beta-secretase and presenilin-1 requires APP. Nature.

[58] Gómez X., Sanon S., Zambrano K. (2021). Key points for the development of antioxidant cocktails to prevent cellular stress and damage caused by reactive oxygen species (ROS) during manned space missions. Microgravity Sci. Tech..

[59] Lesné S., Sherman M., Grant M., Kuskowski M., Schneider J., Bennett D. (2013). Brain amyloid-β oligomers in ageing and Alzheimer’s disease. Brain.

[60] Cohen T.J., Guo J.L., Hurtado D.E., Kwong L.K., Mills I.P., Trojanowski J.Q., Lee V.M. (2011). The acetylation of tau inhibits its function and promotes pathological tau aggregation. Nat. Commun..

[61] Irwin D.J., Cohen T.J., Grossman M., Arnold S.E., Xie S.X., Lee V.M.Y., Trojanowski J.Q. (2012). Acetylated tau, a novel pathological signature in Alzheimer’s disease and other tauopathies. Brain.

[62] Sharma K. (2019). Cholinesterase inhibitors as Alzheimer’s therapeutics (Review). Mol. Med. Rep..

[63] Méndez M., Méndez-López M., López L., Aller M.A., Arias J., Arias J.L. (2011). Acetylcholinesterase activity in an experimental rat model of Type C hepatic encephalopathy. Acta Histochem..

[64] Elufioye T.O., Chinaka C.G., Oyedeji A.O. (2019). Antioxidant and anticholinesterase activities of macrosphyra longistyla (dc) hiern relevant in the management of Alzheimer’s Disease. Antioxidants.

[65] Ikonomovic M.D., Mufson E.J., Wuu J., Bennett D.A., DeKosky S.T. (2005). Reduction of choline acetyltransferase activity in primary visual cortex in mild to moderate Alzheimer’s disease. Arch. Neurol..

[66] Jamshidnejad-Tosaramandani T., Kashanian S., Babaei M., Al-Sabri M.H., Schiöth H.B. (2021). The potential effect of insulin on AChE and its interactions with rivastigmine in vitro. Pharmaceuticals.

[67] Perera T.D., Dwork A.J., Keegan K.A., Thirumangalakudi L., Lipira C.M., Joyce N., Lange C., Higley J.D., Rosoklija G., Hen R. (2011). Necessity of hippocampal neurogenesis for the therapeutic action of antidepressants in adult nonhuman primates. PLoS ONE.

[68] Anschev A.S. (2020). Small intestine acetylcholinesterase activity in experimental animals exposed to low doses of ionizing radiation. Journal of Education. Health Sport.

[69] Dułak D., Gadzała M., Banach M., Roterman I., Irena R.-K. (2020). Analysis of alternative conformations of the AΒ(1–40) amyloid protein. From Globular Proteins to Amyloids.

[70] Xiang Y., Bu X.L., Liu Y.H. (2015). Physiological amyloid-beta clearance in the periphery and its therapeutic potential for Alzheimer’s disease. Acta Neuropathol..

